# Diagnostic performance of focused cardiac ultrasound performed by emergency physicians for the assessment of ascending aorta dilatation and aneurysm

**DOI:** 10.1186/2036-7902-7-S1-A12

**Published:** 2015-03-09

**Authors:** P Nazerian, C Gigli, A Pavellini, FR Ermini, G Pepe, S Vanni, S Grifoni

**Affiliations:** 1 Emergency Department, Careggi University Hospital, Italy

## Background

The diagnostic performance of transthoracic focus cardiac ultrasound (FoCUS) performed by emergency physician (EP) to estimate ascending aorta dimensions in the acute setting is not known.

## Objective

We investigate the accuracy and the intervariability of EP-performed FoCUS to estimate thoracic aortic dilation and aneurysm compared with computed tomography angiography (CTA) as the reference standard.

## Patients and methods

This was a prospective single-centre cohort study of a convenience sample of patients that underwent CTA in the emergency department for suspected aortic pathology. FOCUS was performed before CTA and the maximum ascending aorta diameter measured in parasternal long-axis view. Diagnostic accuracy of FOCUS for detection of aortic dilation (diameter ≥40 mm) and aneurysm (diameter ≥45 mm) were calculated considering CTA as reference standard. In a subgroup of patients, a second EP-sonographer performed FoCUS to evaluate interobserver agreement for the diagnosis of ascending aorta dilation.

## Results

140 patients were enrolled in the study. Ascending aorta dilation and aneurysm were detected at FoCUS in 50 (35.7%) and in 27 (17.8%) respectively. Sensitivity and specificity of FOCUS for ascending aorta dilation were 78.6% (95% CI 65.6-88.4) and 93.9% (95% CI 85.1-97.3) respectively and for ascending aorta aneurysm were 64.7% (95% CI 46.5-80.2) and 95.3% (95% CI 89.3-98.4) respectively. Inter-observer agreement of FoCUS was k = 0.82.

## Conclusions

FoCUS performed by EP showed a good specificity and suboptimal sensitivity for ascending aorta dilation and aneurysm when compared to CTA and appears as a highly reproducible technique.

